# Patient and public involvement in the development of the digital tool MyBoT to support communication between young people with a chronic condition and care providers

**DOI:** 10.1111/hex.14003

**Published:** 2024-03-05

**Authors:** Femke van Schelven, Mara van Weele, Eline van der Meulen, Elise Wessels, Hennie Boeije

**Affiliations:** ^1^ Department Perspective of Patients and Clients in Healthcare Nivel, The Netherlands Institute for Health Services Research Utrecht The Netherlands; ^2^ JongPIT Amsterdam The Netherlands; ^3^ Department of Gastroenterology and Hepatology Amsterdam University Medical Center Amsterdam The Netherlands

**Keywords:** body mapping, co‐design, patient participation, patient‐centred care, shared decision making, telemedicine, young people with a chronic condition

## Abstract

**Introduction:**

To guide good practices in patient and public involvement (PPI), several calls have been made to share detailed accounts of practical experiences. We describe our collaboration with young people with a chronic condition (YPCC) in the development, testing and implementation of the digital communication tool MyBoT (Map your Burden of Treatment).

**Methods:**

MyBoT was developed by a team of academic researchers, some of whom were practising care providers, YPCC and designers. In addition to the two co‐researchers in the research team, various groups of YPCC were involved in decision‐making through participation in a design session, workshops and a dialogue session. The Involvement Matrix was used to reflect on the PPI of all YPCC.

**Results:**

Initially, the two co‐researchers were involved in the roles of informer and co‐thinker, but their decision‐making power within the study increased over time. In the final stages of the study, the co‐researchers and researchers became partners. The other YPCC who participated in the different sessions and workshops were co‐thinkers in all stages of the study.

**Conclusion:**

The PPI of two YPCCs as co‐researchers within the research team ensured continuous involvement, whereas the PPI of various groups of YPCCs guaranteed a representative and inclusive approach. Researchers play an essential role in bringing all perspectives together, integrating them within the technical and financial constraints and ultimately building a tool that is tailored to its users' needs.

**Patient or Public Contribution:**

YPCC played a significant role in the present study. Two YPCC—who are also co‐authors of this paper—were involved in all stages of this project as members of the research team. In addition, various YPCCs were involved in the development, testing and implementation stage of MyBoT by organizing design sessions, workshops and a dialogue session.

## 1 INTRODUCTION

Research is increasingly carried out together with young people with a chronic condition (YPCC) rather than only for or about them.^1^, ^2^ This collaboration with (young) patients in research is termed patient and public involvement (PPI)^3^ and is driven by a strong belief that it contributes to the quality and relevance of research.^4^, ^5^ Additionally, PPI does justice to the right of young people to have a say in matters that affect them, as is enshrined in the United Nations Convention on the Rights of the Child.^6^

Patient and Public Involvement can take many forms. YPCC can contribute to research in many ways, for instance, as co‐researchers who are part of the research team,^7^, ^8^ as members of an advisory panel^9^ or as participants in co‐creation workshops.^10^ Their roles and decision‐making power in different stages of research can vary as well. Based on the work of Arnstein on citizen participation,^11^ Hart has developed the Ladder of Involvement, which distinguishes eight roles in which young people have different degrees of agency when working together with adults.^12^ These roles vary from (undesirable) tokenistic roles to roles in which young people are informed and consulted by adults and roles in which they have actual decision‐making power within research.

PPI is a context‐dependent and dynamic process.^13^ It is not a simple intervention, and there is no one‐size‐fits‐all approach.^14^, ^15^, ^16^ What is considered the best way to work together depends on several factors, such as the YPCC who are involved, the type of research and the context the research takes place in.^1^, ^16^ To ensure that all roles and responsibilities match with those involved, PPI processes are shaped gradually during the research and in dialogue between YPCC and researchers.^14^, ^15^ It is seldom clear in advance how PPI processes will develop. This is at odds with the way most research is conducted, and therefore, researchers are struggling with how to shape their collaboration with YPCC.^17^

To guide good practices in PPI with different patient groups, calls have been made to share detailed accounts of practical experiences with PPI.^15^, ^18^, ^19^, ^20^ In this paper, we will therefore extensively describe our collaboration with YPCC in the development, testing and implementation of the communication tool MyBoT (*M*ap *y*our *B*urden *o*f *T*reatment) for YPCC and healthcare providers. The tool addresses treatment burden, which concerns the physical and emotional side‐effects of treatment and the perceived weight of actions and resources (young) patients devote to their healthcare, such as time dedicated to medication and self‐monitoring.^21^, ^22^, ^23^ Generally, limited attention is paid to the social and spiritual challenges of treatment adherence in the communication between YPCC and their care providers due to people's hesitation to share treatment experiences in the presence of an authority figure^24^, ^25^ and care providers' tendency to focus on physical symptoms and side‐effects.^25^, ^26^

The form and content of the MyBoT tool are based on the visual method of body mapping. This method enables visual representation of people's lives, their bodies, the world they live in and the way these are connected. In body mapping, a life‐sized outline of someone's body is traced, which is then populated with visual representations, symbols and words related to the topic under study.^27^, ^28^ In the words of de Jager et al., a body map communicates a message such as ‘This is who I am, this is my story, and this is what is important to me’.^27^ Several studies have pointed out that body mapping provides rich insights into lived experiences and social contexts.^27^, ^28^, ^29^ It has benefits for those who participate in body mapping activities, such as increased awareness and greater understanding of the richness of their life stories and a greater sense of self‐worth, power and agency.^27^, ^28^

To ensure that MyBoT matched daily practice and the wishes and needs of end‐users, it was developed in close collaboration with various groups of YPCC.^30^, ^31^ In this paper, we aim to contribute to the field of PPI by sharing and describing our experiences and the lessons learned. We will answer the following research question: How were YPCC involved in the development, testing and implementation of the digital body map tool MyBoT, and how did their involvement influence decisions?

## 2 METHODS AND FINDINGS

MyBoT was developed by a team of five academic researchers (two of whom were also practising care providers) and two YPCC. It was programmed by a design studio that develops serious games to improve health and care. MyBoT can be used on the computer and smartphone and is freely available online: https://app.whappbot.com/chat/bodymap. The language used is Dutch.

The MyBoT study took place in four stages: (1) project idea and proposal; (2) tool development; (3) application and testing of the tool and (4) implementation. PPI was a core element of the study, and YPCC played different roles with various degrees of decision‐making power in each stage. PPI took place in two ways. Two YPCC (E. M. and E. W.) were members of the research team that created MyBoT, and several other YPCC were involved by participating in group sessions and workshops that informed tool development.

**Figure 1:**
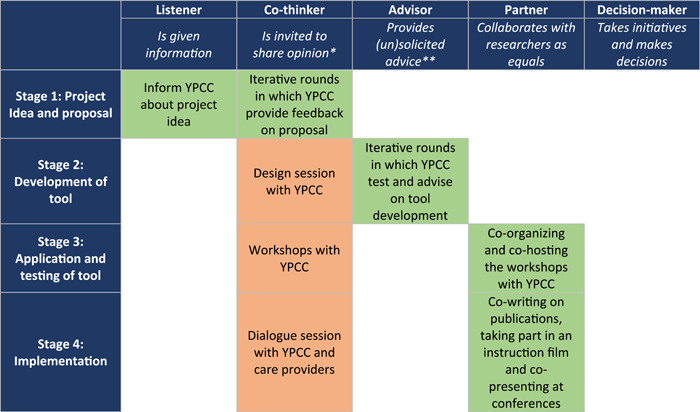
Visual representation of PPI in the MyBoT study—based on the Involvement Matrix.^18^ The green cells represent the roles of the two YPCCs who were members of the research team, and the orange cells the roles of the various YPCCs who were involved in the various group sessions and workshops. *Researchers decide whether to use it and how; **solicited advice is binding. Unsolicited advice must be given formal attention and may only be rejected with substantiated arguments. MyBoT, Map your Burden of Treatment; PPI, patient and public involvement; YPCC, young people with a chronic condition.

We have used the Involvement Matrix to evaluate the roles YPCC has played in the MyBoT study (Figure 1). Although the Involvement Matrix originally is a tool to facilitate PPI processes and the dialogue about the roles of patients in (research) projects,^18^ such tools have also proven to be valuable in evaluating PPI at the end of a study.^20^, ^32^ The Involvement Matrix is based on Arnstein's^11^ and Hart's^12^, ^33^ work on PPI and helps to map PPI activities during different project stages. We have put the four stages of the MyBoT study on the vertical axe of the matrix. On the horizontal axe are the roles YPCC can play, including ‘listener’, ‘co‐thinker’, ‘advisor’, ‘partner’ and ‘decision‐maker’. The combination of the stages and roles results in a matrix with cells, in which we have summarized the PPI of YPCC during each stage. In the remainder of this section, we describe the PPI activities in the four stages in more detail.

### 2.1 Stage 1: Project idea and proposal

The first stage of the study aimed to write a project proposal and obtain funding. YPCC were involved in this stage in the roles of listener and co‐thinker. The activities in this first stage resulted in a project proposal, which was accepted for funding.

#### 2.1.1 Writing the project proposal

Two YPCC from the network of JongPIT, a Dutch organization for and led by YPCC, were recruited to help translate ideas about a study on body mapping and treatment burden into a project proposal. They responded to an invitation letter from the researchers containing information about the initial study ideas and the possible roles YPCC could play in writing the project proposal. In a physical meeting, the researchers and YPCC got acquainted with each other and discussed the project proposal. Initially, they planned to develop a physical body map tool that YPCC and care providers could use to draw a body map and discuss treatment burden. The two YPCC involved in the study were enthusiastic and highlighted the importance of using visual methods in the tool to ensure the project would not result in another questionnaire YPCC had to fill out before or after consultations with their care provider. They also had practical suggestions about the way the research activities described in the project proposal could be set up to ensure that YPCC felt safe and supported to share their visions. Through phone calls and emails, the YPCC provided feedback on several versions of the project proposal until the last version was finalized. As both YPCC wished to remain involved in case the project proposal would be accepted, they were added to the proposal as research team members.

#### 2.1.2 Adapting the project proposal after the outbreak of COVID‐19

The project proposal was accepted, and the study started at the beginning of 2020. Preparations were made to initiate live body mapping sessions using drawing and painting. However, these plans had to be adjusted due to the outbreak of COVID‐19. Live sessions were no longer possible due to restriction measures to prevent the spreading of the disease. In consultation with the research team and grant commissioner, it was decided to adapt the project proposal and create a digital body map tool for YPCC and their care providers that could be used without in‐person meetings. To this end, the research team of researchers and YPCC was extended by adding developers from a design studio.

### 2.2 Stage 2: Development of tool

The second stage of the study focused on developing the digital body map tool. YPCC were involved as co‐thinkers and advisors. The activities in this stage resulted in a digital demo version of the tool.

#### 2.2.1 Identification of relevant topics

Based on a quick scan of the literature on young people's experiences with treatment burden, the research team devised a list of topics that were relevant to address in the digital body map tool MyBoT. An interview with a psychotherapist who worked with young cancer patients was conducted to identify additional topics. This interview addressed the course of consultations and impeding and facilitating factors for dialogue about treatment burden. Amongst other things, the care provider emphasized that YPCC need to learn to take initiative in discussing issues that matter to them. Therefore, we refined the topic list and added a topic that encouraged YPCC to write down issues they wished to discuss during their next consult. After finalizing the topic list, we used scientific and grey literature on body mapping to translate the collected topics into questions that could guide young people in creating their body maps. These questions were structured into a script (Appendix SA).

#### 2.2.2 Development and testing of paper prototype

**Figure 2:**
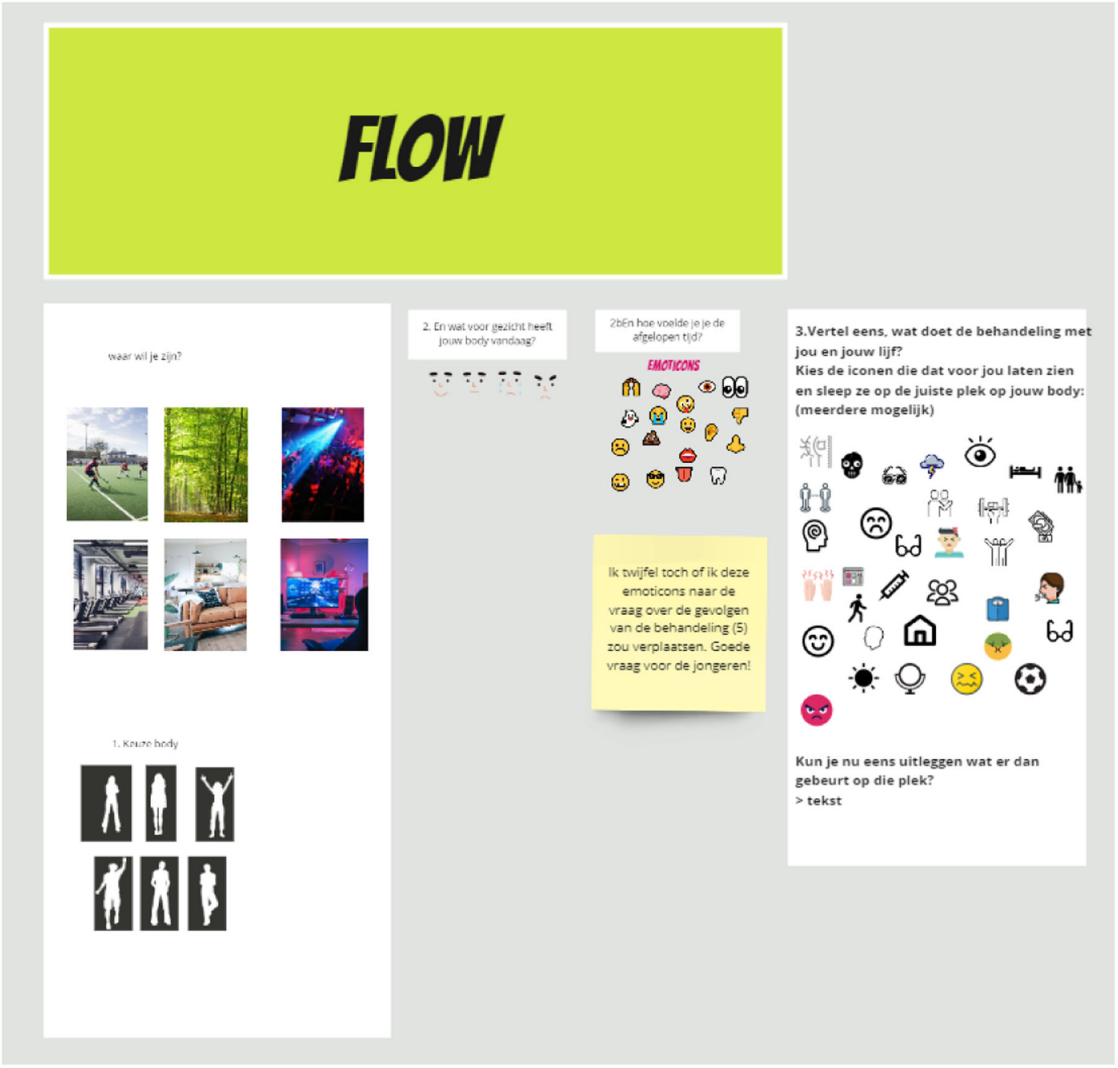
This is the first part of the paper prototype. The first text box on the left contains the text ‘Where do you want to be?’ and ‘Choice of body’, including various options for visuals. The second text box reads, ‘What facial expression does your body have today?’ and the third ‘How have you been feeling lately?’. On the post‐it below the third text box, a note was placed by one of the researchers saying, ‘Maybe we should move these visuals to the question about the consequences of treatment. We should ask the young people!’. The text box on the right contains the text ‘Tell me, what effects does treatment have on you and your body? Pick the visuals that show this and drag them to the right place on your body map’, followed by ‘Can you explain what happens on this place [on your body]?’.

Based on the script, the design studio created a paper prototype: an initial, substantive design of the tool consisting of a flowchart of the questions that guide young people through the body mapping process. Figures, icons and pictures were designed to be used to create a body map. Figure 2 shows the first part of the flowchart to provide an idea. The paper prototype followed the prepared script and started with building the base of a body map, i.e. choosing a background, body pose and face. Questions such as ‘where do you want to be?’ (for the background) and ‘how do you feel?’ (for the facial expression) guided this. Then, questions were asked to stimulate reflection and help fill the body map with icons, photos and text. Questions addressed the well‐being of YPCC, the physical and psychosocial impact of treatment, social support and coping strategies. The paper prototype finishes with a question about the specific questions YPCC wishes to ask their care provider.

The paper prototype was tested in a co‐design session with YPCC. This session was hosted by two developers from the design studio and a researcher (F. S.). Four young people (16–25 years) who underwent treatment for a chronic somatic condition participated. They were recruited through an invitation on the website of JongPIT. At the start of the session, the hosts explained the aim of the project and the concept of body mapping (Appendix SA). They invited the participants to build a body map based on the questions and visuals in the paper prototype. They worked on Miro‐Board, an online whiteboard tool, using post‐its and visuals such as body poses, facial expressions, icons and emoticons. Participants found body mapping an appropriate way to share their experiences about treatment burden. They noted it could help them to focus on topics that mattered to them during consultations with care providers. This validated the choice of body mapping to help YPCC to discuss the treatment burden.

The participants of the co‐design session suggested several adaptations to the paper prototype. For example, they proposed to include bodies in wheelchairs in addition to the standing bodies that were initially offered to them. They also wished for the opportunity to pick two facial expressions. This would enable them to show the contrast between how they feel and how they pretend to feel to the outside world. The participants noticed the questions predominantly focused on negative issues. They liked to add positive aspects as well since this sometimes motivated them to choose a specific treatment. The YPCC in our research team confirmed this. The participants of the co‐design session also provided suggestions regarding some technical issues, such as the possibility of putting personal photos on the body maps, being creative, drawing and using words to view their progress in the body mapping process and to be able to go back to make changes and to save their work.

#### 2.2.3 Programming the digital tool

The final paper prototype was used to programme the digital version of MyBoT. This was an iterative process. The design studio built a demo version on a software platform that enables (written) interactions with a chatbot. They also created visuals needed for body mapping, such as backgrounds, bodies, facial expressions, and icons. The research team composed a text for the chatbot to introduce the tool, treatment burden and body mapping and to guide YPCC in making a body map. We considered a clear and empathetic formulation of the questions and responses of the chatbot important to create a safe environment for sharing potentially sensitive topics as well as language that fits the lifeworld of YPCC. Below, we provide two examples of interactions that tool users may have with the chatbot.

Example 1: Fictitious chat interaction with the chatbot

CHATBOT: To make it a bit more personal: How would you name your body map? Please use a nickname;

TOOL USER: ResearchTeam

CHATBOT: Nice to talk to you, ResearchTeam!

Take a look at the faces below. Which face best describes how you feel today?

TOOL USER clicks on the happy face.

CHATBOT: Looks like you're feeling good today! Where can I put this on your body map?

Example 2: Fictitious chat interaction with the chatbot

CHATBOT: Is there an activity you really want to do but can't because of your treatment? Do you have a photo of that?

TOOL USER browses computer for a photo.

CHATBOT: Where on your body map do you want to place this photo?

TOOL USER clicks on the desired location.

CHATBOT: Why is this activity not possible for you?

TOOL USER provides an open answer.

CHATBOT: These questions are not always easy. Thank you for your answers, ResearchTeam.

The suggestions YPCC had made during the co‐design sessions were incorporated as much as possible in the digital tool. Different functionalities were incorporated, such as the possibility to go back and make changes to the body map and add personal photos. More diverse bodies were developed, including those in wheelchairs, and we made sure the chatbot also asked about the positive aspects of treatment. However, due to restraints in technical and financial resources, not all wishes could be taken up fully. It was, for instance, not possible to add a drawing option.

#### 2.2.4 Iterative rounds of testing

The research team provided feedback on multiple versions of the demo. Based on the input collected from YPCC so far, the research team formulated essential criteria for MyBoT. The tool should (a) look mature and serious, (b) be clear and professional, (c) facilitate young people to create body maps by themselves on their computer, (d) facilitate the use of both visuals and words, (e) not be too time‐consuming and (f) ensure security and privacy of its users. Taking into account these criteria, the tool was further refined. Improvements were especially made in the area of criteria (b) and (e), as the first version of the tool lacked user‐friendliness. For example, in the first version of the digital demo, visuals could only be placed on the body map by typing and sending a number to the chatbot that corresponded to a specific place on a grid that was placed over the body map. This was deemed inconvenient by all research team members. The most ideal solution (drag and drop) to put visuals in the right place in the body map was not possible due to technical and financial constraints. The research team, therefore, searched for alternatives and eventually chose to make the grid clickable and more detailed.

**Figure 3:**
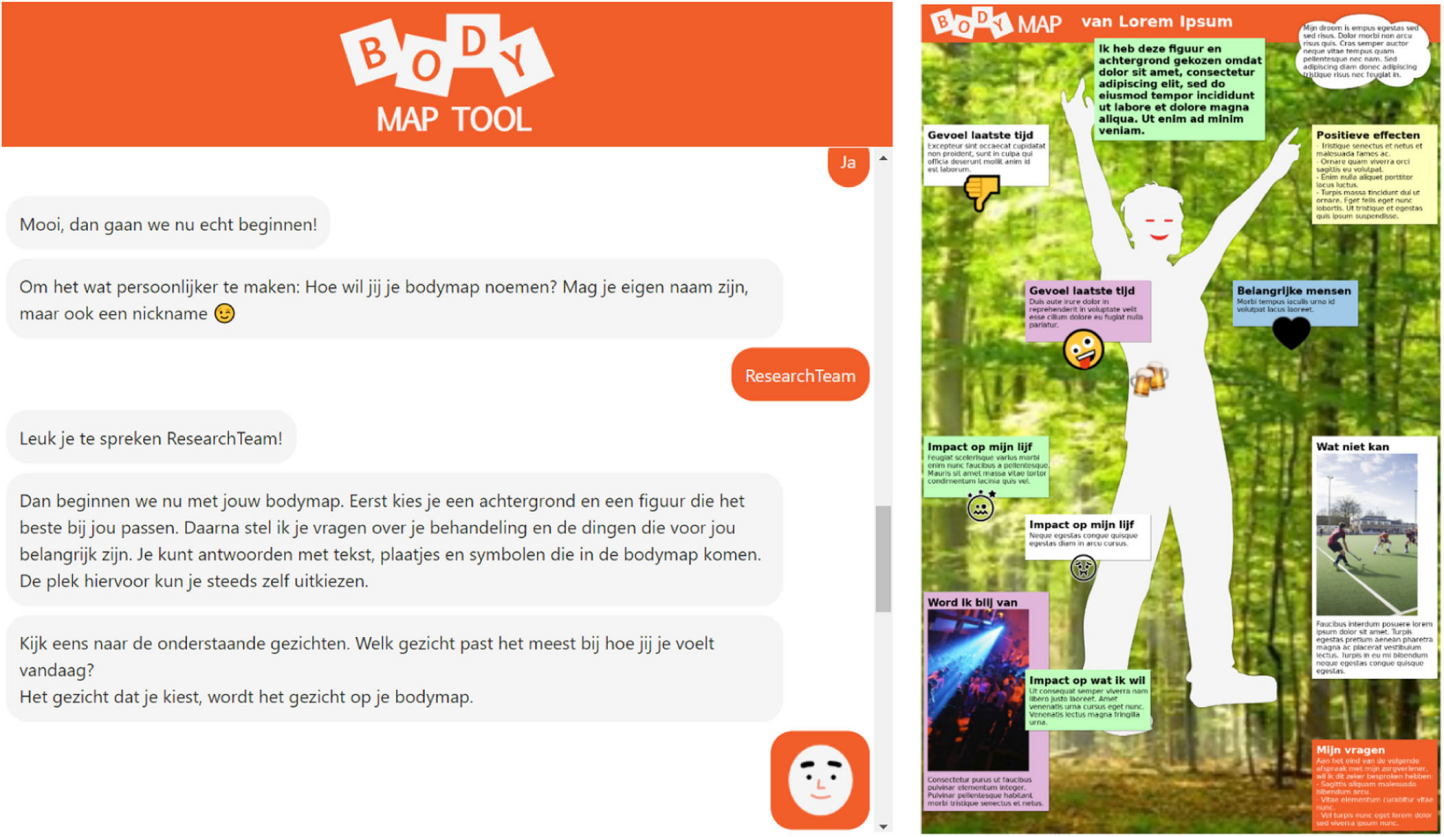
These screenshots depict a fragment of a conversation with the chatbot in Dutch (the language used in the tool) and a fictional example of a body map. In the fragment, the chatbot initiates the body mapping process and asks for the user's nickname. Then, it provides a brief introduction to the body mapping process and invites the user to select a facial expression for the body map. The fictional example shows how users can put icons and photos on the body map and use text to explain them. Texts can relate to different topics (described in the headings of the text boxes), such as the positive effects of treatment, the impact of treatment on the body, emotions, the impact of treatment on meaningful activities, coping strategies and important people and dreams.

The resulting digital demo was a version that covered the input from YPCC as much as possible and did justice to the aim of the body mapping method within the possibilities regarding finances and manpower. By answering questions from the chatbot, tool users could choose visuals, place them in various places on their body map and—if desired—provide a textual explanation. Figure 3 is a screenshot from the digital tool, with the chatbot on the left and the resulting body map on the right.

### 2.3 Stage 3: Application and testing of the tool

The third stage focused on using the digital demo with YPCC to discuss and learn about treatment burden and, at the same time, evaluate the digital tool. YPCC were involved as co‐thinkers and partners. This stage resulted in the final version of the MyBoT tool.

#### 2.3.1 Digital body mapping workshops

Two series of three body map workshops were organized. Young people (16–25 years) who underwent treatment for a chronic somatic condition were invited to participate. They were recruited through an announcement placed on the website of JongPIT. In total, 10 young people participated (Table 1). They had different chronic conditions, such as diabetes, rheumatoid arthritis and cerebral palsy.

**Table 1:** Participants in the workshops.

	Participants
Total (*N*)	10
Gender (*n*)	
Female	7
Male	3
Age (years)	
Mean	21.7
Minimum–maximum	17–25

Two groups of four and six young people participated in three workshops of one and a half hours. All workshops were prepared and hosted by one researcher (F. S.) and one adolescent with a chronic condition (E. M. or E. W.). During the online workshops, young people created a body map using MyBoT. Guided by the hosts, they went through the tool step‐by‐step, discussing the choices they made. The hosts asked questions to stimulate reflection on the body maps (see example below). The protocol of each of the workshops is described in Appendix SB. Workshops were audiotaped and transcribed verbatim.

Example: Probing questions stimulating the body mapping process and reflection

1. What body position or posture did you choose and can you tell us more about that?
2. Can you also tell us something about how you portrayed your face?
3. Why did you put this icon in this specific place?
4. What does this photo mean to you?
5. Why did you choose to portray the people around you with this icon?
6. What new insights did your body map give you?

In the first workshop, specific attention was paid to establishing a secure environment in which YPCC could share sensitive matters. This was achieved by allocating considerable time for introductions, facilitating online participation with the webcam turned off and adopting a personal approach. After the introductions, participants went through the initial steps of the tool to start their personal body map, namely a body, facial expression and background. They also added some icons to their body map by answering questions about their well‐being and the impact of treatment on their body. In the second workshop, participants continued to fill their body map with icons, photos and text by answering questions about the activities they like to do now and in the future, the impact of their treatment on these activities and the positive impact of their treatment. In the third workshop, participants added icons and text to their body maps that addressed coping strategies and aspects of treatment they wished to change and discuss with their caregivers.

The direct and indirect feedback of the participants in the workshops resulted in final adaptations of the tool. Regarding the visuals, participants asked for more diverse bodies with more body weight as they argued that treatment sometimes results in weight gain. They also wished for clearer facial expressions since they found it difficult to distinguish the emotions of the current expressions. Due to the chosen colours, some of the icons disappeared into the background of the body maps. All suggestions regarding the visuals were taken up. Other changes to the tool were made as well. We noticed that some questions were not fully understood by all participants. It was, for instance, not always clear that the questions concerned treatment burden and not the burden of disease. We clarified this by formulating questions in more detail and stating clearly that the questions focused on treatment. The participants also noted that an overload of visuals made their body maps too full and crowded. Therefore, we limited the number of visuals that could be selected for each question. Finally, participants reported that they liked it when the chatbot said something humane, such as ‘Thank you for being so open to me’. This helped to answer questions about sensitive topics. We added this type of sentence more frequently.

### 2.4 Stage 4: Implementation

The final stage focused on implementation possibilities for MyBoT. YPCC were involved as a co‐thinkers and partners. The stage resulted in several activities to promote future implementation.

#### 2.4.1 Dialogue session with YPCC and care providers

We organized a dialogue session with YPCC and care providers. Young people (16–25 years) who underwent treatment for a chronic, somatic condition were recruited through an announcement placed on the website of JongPIT. Care providers who treated YPCC were recruited through the networks of the research team. They were approached via email and a post on social media. In total, five YPCC and six care providers—a rheumatologist, paediatric neurologist, two psychologists and two youth coaches—participated. The dialogue session lasted 2 h and was hosted by two researchers (F. S., H. B.). It started with an introduction about treatment burden and body mapping. After getting to know each other, the hosts presented findings from the workshops described in the previous step and showed MyBoT to participants. The hosts facilitated a dialogue between participants about the use of the tool and how it could be applied in practice.

The participants in the dialogue session found MyBoT of added value in the communication between YPCC and care providers. YPCC said that they always had to fill out a lot of questionnaires, and they liked that MyBoT was different. Care providers indicated that body mapping encourages reflection and, in doing so, adds elements to communication as usual. This communication focuses on medical topics, while body mapping gives rise to discussions about other areas of life as well, such as relationships, education, hobbies and the future, which matter a lot to young people. This provides insights into the considerations and behaviour of YPCC regarding treatment adherence, which can now become part of making decisions together. Care providers also reported that body mapping with MyBoT could facilitate an equal dialogue between them and young patients. The young people who participated in the dialogue session confirmed this and said that dialogue would also increase their trust in care providers.

We also discussed situations in which MyBoT may be of added value. YPCC and care providers agreed that it can be helpful in different phases of treatment and life. Just after diagnosis, it can be valuable to make a body map about treatment burden experiences, but also, in later stages, it can be helpful to reflect on the ways treatment interferes with life goals. Care providers highlighted that MyBoT could be helpful in evaluating treatment burden during life events—such as finishing school and starting a job, moving out of the parental home or transitioning to adult care—as new questions may arise.

Some facilitating and hindering factors for implementing the digital tool came up as well. It was considered an advantage that the tool is freely available online to both YPCC and care providers. Consequently, its use does not depend on one party: they can both take initiative. Lack of time among care providers was considered the biggest hindering factor for the implementation of the tool. Another hindering factor was the dependency on care providers' conversation skills. Although the chatbot guides the body mapping process, MyBoT can only be successful when care providers are willing and able to discuss body maps. Finally, participants considered body mapping a time and energy‐consuming process. However, they also acknowledged that good reflection takes time.

#### 2.4.2 Implementation and dissemination activities

The research team undertook several activities to disseminate MyBoT. Options and considerations for using the tool were described in a guiding (Dutch) publication. The YPCC advised on the content of this publication and wrote 10 tips on how to use MyBoT. Box 1 provides a short summary of how MyBoT can be used in clinical practice. A short instruction movie was recorded with a researcher (F. S.) and a YPCC (E. W.) explaining the tool. MyBoT and the movie were placed on a webpage about the tool, which was shared on different websites addressing young patients and care professionals. The webpage was actively promoted in a network of the research team, and researchers and YPCC presented the tool at several conferences.

BOX 1 **Utilization of MyBoT in clinical practice**

To ensure the suitability of MyBoT across different settings, its utilization offers flexibility. YPCC have the option to employ it based on the advice of care providers, but they can also suggest it themselves. As the body mapping process is facilitated by a chatbot, YPCC can collaboratively create their body map with a care provider or undertake the task individually. The creation of their body map facilitates reflection on the treatment burden, motivating them to consider both bottlenecks and opportunities. Given the personal and sensitive nature of the information in body maps, YPCC retain the autonomy to decide whether to show it to their care provider. Another option is to write down questions and insights that emerged during the creation of the body map. During consultations, the body map can serve as a focal point of discussion, enabling YPCC to communicate about the impact of treatment on daily life, discuss bottlenecks in adherence and engage in shared decision‐making with care providers.

## 3 DISCUSSION

The present study described the involvement of YPCC in the development, testing and implementation of MyBoT, a digital body map tool that supports and stimulates communication between YPCC and care providers about treatment burden. By providing a detailed account of the ways YPCC was involved and influenced decisions about this digital tool, we aim to contribute to the broader PPI field and inspire future PPI practices with (young) patients. In Box 2, we have summarized the most important practical lessons learned.

BOX 2 **Lessons learned in the MyBot study about PPI of YPCC in digital tool development**

1. YPCC can and should be involved in tool development studies to ensure the tool matches their life worlds. A tool is a tangible product and YPCC have extensive ideas on how to shape, develop and use it.
2. Based on needs and preferences, the roles YPCC play in tool development can vary per individual, such as co‐thinker, advisor and partner.
3. The roles of individual YPCC in tool development vary throughout the study based on changing needs and preferences and the content of specific project stages.
4. Balance different forms of involvement to realize different purposes, for example, continuous involvement as co‐researchers enables a limited number of YPCC with inside knowledge to assume diverse roles, including those with more decision‐making power, and occasional involvement through workshops and creative sessions, enables the inclusion of a diverse group to realize representation of different life worlds.
5. The greater decision‐making power of researchers as project administrators mainly places the responsibility on them to take efforts to include YPCC by communicating continuously, listening attentively, and incorporating and discussing their input.
6. PPI activities in tool development can successfully take place online to replace in‐person presence and facilitate the inclusion of introvert YPCC or YPCC who are unable to join physically.
7. The utilization of visual methods in PPI activities, such as mood boards and whiteboards, facilitates free thinking and associating of YPCC in tool development.

We have used two forms of PPI in our study: (1) two YPCC were involved in all stages of this project as members of the research team and co‐authored this paper and (2) various YPCC participated in specific stages of the project, i.e. the design session, workshops and dialogue session. This dual approach ensured both continuous involvement and involvement of a broad group of young people. As can be seen in the Involvement Matrix (Figure 1), the two co‐researchers generally had greater power to influence decision‐making compared to the YPCC involved through the sessions, and their decision‐making power increased over time. Confirming that PPI processes seldom developed according to a predetermined plan,^15^, ^32^ the involvement of the co‐researchers exceeded initial expectations. At the start of the study, most initiatives for involvement came from the researchers, who were inviting the co‐researchers to contribute. This gradually grew into a partnership, as their continuous involvement enabled the co‐researchers to become familiar with the study and develop ownership of the tool and study. Other factors that contributed to their growing involvement were the enthusiasm and dedication to the topic of the co‐researchers and the efforts of the researchers to involve them in every aspect of the study, which included continuous communication and attentively listening, discussing and incorporating contributions made by the co‐researchers.^14^, ^20^, ^34^

The YPCC who participated in the co‐design session, workshops and dialogue session had the role of co‐thinkers in all stages of the study. These YPCC provided input for the development and implementation of the tool, and the research team subsequently decided whether and how this was incorporated into the tool. An advantage of this form of PPI lies in the facilitation of a greater and more varied group of YPCC alongside the co‐researchers. This proved especially instrumental in the first stages of the study, for example, in exploring topics, testing chat texts, balancing visuals and text, and writing empathic reactions within the chat. A significant challenge, however, was the lack of familiarity of these YPCC with the study, necessitating comprehensive explanations. Effectively communicating the idea of a digital body map tool about treatment burden without unduly influencing their thoughts and suggestions presented a dilemma. We aimed for YPCC to freely associate and think independently. The utilization of the Miro‐board during the design session emerged as a valuable strategy, mitigating the need for extensive verbal explanations. The visualized paper prototype helped YPCC to freely associate and think independently.

This paper has described in detail how YPCC influenced decisions about MyBoT. They were, however, not the only stakeholders in the development process. Researchers, care providers and designers were involved as well. Consistent with previous research, we found that the diverse backgrounds and needs of all stakeholders—including scientific and experiential knowledge of treatment burden, healthcare, body mapping and tool usage—were crucial and distinctive in their contributions to the project.^35^, ^36^ While the interests of the stakeholders generally overlapped, conflicts arose in some areas. YPCC and care providers often expressed their preferences for features that promoted creativity and flexibility in the body mapping process such as functionalities that enable drawing, whereas the designers considered technological feasibility and cost considerations. In addition, we noticed that care providers often tended to think in existing frames and protocols, such as diagnostic tests that can be repeated over time, while the development of the body map tool required thinking in new opportunities with a visual tool. Previous studies have also reported conflicting interests between users, designers and researchers and emphasized that generally, not all preferences can be met, as this may lead to a hybrid tool that is unacceptable to everyone.^36^, ^37^ These studies also emphasize that attention should be paid to the power imbalance that may exist in PPI processes, especially between YPCC and the other stakeholders. In the present study, we experienced that the researchers on the project team played a key role in collecting all perspectives, bringing them together, weighing them and designing a final tool that meets project requirements through collaboration with its users.

### 3.1 Strengths and limitations of the study

The present study provides a detailed account of the ways YPCC could be involved in the development, testing and implementation of a digital tool. It is authored by both researchers and YPCC, ensuring the inclusion of different perspectives in the descriptions. A limitation of our involvement approach concerns the diversity of the YPCC involved. They were mostly female and relatively highly educated; none of them had a migration background. COVID‐19 posed another important limitation to the study, as it required major changes to the initial plans. The flexibility of the research team enabled collaboration and PPI on a distance— using mail, phone and video calling—and led to an innovation in the healthcare field: a tool that enables digital body mapping guided by a chatbot. The available means were another limitation of the study since this sometimes restricted the possibilities for following the suggestions provided by YPCC. We discussed these restrictions with the YPCC and involved them in prioritizing all input.

## 4 CONCLUSION

The present study answers calls for detailed descriptions of PPI to improve our understanding of the ways (young) patients can be involved in research. We have shown that applying multiple forms of PPI has important benefits. The involvement of two YPCC as co‐researchers within the research team ensured continuous involvement, whereas the involvement of various groups of YPCC guaranteed a more inclusive approach. Employing the Involvement Matrix as a reflective tool, we observed that continuous involvement led to a significant advantage by fostering growth and development in their roles and developing ownership. The incorporation of the views of the other YPCC (who were involved through design sessions and workshops) remained more dependent on the researchers. Considerable efforts were dedicated to incorporating all input within the technical and financial constraints, resulting in a tool tailored to the needs of its users.

## AUTHOR CONTRIBUTIONS

**Femke van Schelven**: Conceptualization; data curation; formal analysis; funding acquisition; investigation; methodology; project administration; writing—original draft; writing—review & editing. **Mara van Weele**: Writing—review and editing. **Eline van der Meulen**: Formal analysis; investigation; writing—review and editing. **Elise Wessels**: Formal analysis; investigation; writing—review and editing. **Hennie Boeije**: Conceptualization; data curation; funding acquisition; investigation; methodology; project administration; writing—original draft; writing—review and editing; supervision.

## CONFLICT OF INTEREST STATEMENT

The authors declare no conflict of interest.

## ETHICS STATEMENT

The Medical Ethics Committee Utrecht decided that the Medical Research Involving Human Subjects Act (WMO) does not apply to this study, and therefore, official approval of this study by the METC is not required under the WMO (reference 20‐082/C). All methods were carried out in accordance with relevant guidelines and regulations, for example, the General Data Protection Regulation and informed consent was obtained from all participants.

## Supporting information

Supporting information.

Supporting information.

Supporting information.

## Data Availability

The datasets generated and/or analyzed during the current study are not publicly available due to the small sample size of the study, but deidentified data are available from the corresponding author on reasonable request.
