# Supplementary material for: Patient and public involvement in the development of the digital tool MyBoT to support communication between young people with a chronic condition and care providers
Source: Health Expect. 2024 Mar 5;27(2):e14003. doi: 10.1111/hex.14003 (PMC10915502; doi:10.1111/hex.14003)
Supplement: Supplementary file 1 — Supporting information. [file HEX-27-e14003-s002.docx]

**Appendix A. Program of MyBoT design session**

**Welcome**

The session is hosted by two designers. A researcher is present as ‘fly on the wall’. Four young people with a chronic condition participate. Everyone shortly introduces themselves based on the question: *if you’d know me, you’d know that…*.

**Introduction**

The designers explain the aim of the digital tool that is going to be developed. They invite the participants to help in the creation of the paper prototype of the tool. To this end, the concept of body mapping is shortly explained (i.e. a body map is an image of a body on which symbols, photos and other pictures can be put to visualize treatment burden). To facilitate an open mind among participants, this explanation does not go into detail about the ways treatment burden could be visualized into a body map. This is up to the participants to explore during the session.

**Miro board session**

The designers invite participants to join the Miro board that was set up for the design session. They explain how Miro board works. The designers have prepared a separate, personal space on the Miro board for each participant. Participants have to look for their personal space and can leave their screen on this part of the Miro board during the entire session. The designers ask questions and provide participants with a set of visuals in their personal space that can help them to answer the questions. The visuals are used as building blocks for their body maps.

*Figure A1. Example of the personal space of one of the participants. On the right, questions are posed and visuals are provided. On the left, the body map is built.*


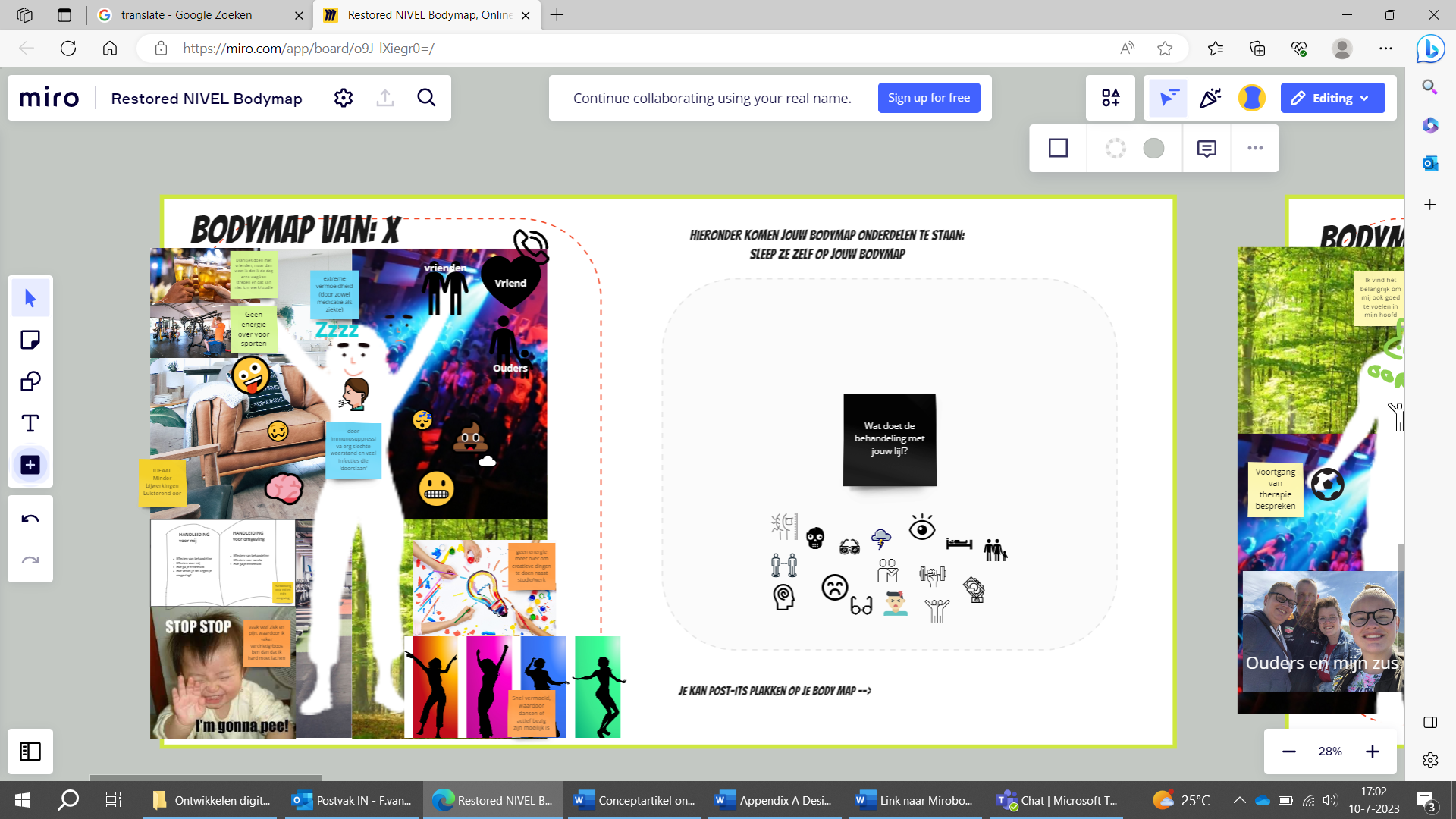


**Script of questions that guided participants**

Below, the questions that guided participants in creating their body maps are described.

Introduction questions to shape the basis of the body map

1. Where would you like to be? (for choosing background of body map)
2. Which body do you chose?
3. What facial expression does your body map have today?

Questions to fill the body map with visuals

1. How do you feel lately?
2. What effects has your treatment on you and your body?
3. Think about the activities that make you happy. Can you tell me something about this activity?
4. How does your treatment affect this activity?
5. Are there any activities you’d like to do but can’t because of your treatment?
6. Does treatment affect other life domains? For example, school, sports, friends and family, jobs?
7. What people are there for you and what do they mean to you?
8. What things do you do to ensure that your treatment fits well into your life?
9. If anything would be possible, what would help you to fit treatment into your life?
10. What is it that you definitely wish to discuss with regard to your future?

**Reflection on digital body mapping**

After each question, participants are invited to reflect on the choices to evaluate whether they understood the concept of body mapping. In addition, they provide feedback on the questions that were asked and the visuals they were provided with to answers the questions. At the end of the session, the overall content of the tool is discussed and whether this requires adaptations.

**End meeting**

The designers and researcher thank the participants for their input.
