# Supplementary material for: Patient and public involvement in the development of the digital tool MyBoT to support communication between young people with a chronic condition and care providers
Source: Health Expect. 2024 Mar 5;27(2):e14003. doi: 10.1111/hex.14003 (PMC10915502; doi:10.1111/hex.14003)
Supplement: Supplementary file 2 — Supporting information. [file HEX-27-e14003-s001.docx]

**Appendix B. Program of body mapping workshops**

**Program of the first workshop (1,5 hours)**

*Welcome and explanation project (15 min)*

- Treatment burden is elaborated on as well as body mapping. The partners of the project are introduced and the researchers and experts by experience.
- During the workshops a demo version of the tool is used. It is promised that at the end of the three workshops everyone has created a body map.
- The duration of the workshop is 1,5 hours and if breaks are needed, that is fairly well possible. Just give us a signal or write it in the chat.
- Possibility of questions is explicitly offered. Informed consent is asked for recording the session.

*Getting to know each other, icebreaker (15 min)*

Participants ask each other one question and take turns, such as what is your best characteristic, which famous person would you like to be for one day, for what can I wake you up at all times.

All participants receive the link of the whappbot in the chat.

*Start of the body mapping (45 min)*

- - Start explaining the tool by looking at an example. Choosing to place something inside or on the body is explained as well as positioning something outside the body. Questions are answered.
  - It is announced that we introduce each step and that we will probably do 4 steps in this first session.
  - All participants chose a name for their body map.
  - Everyone choses a face, a figure and a background.

In between questions are asked to elaborate the choices made: which faces, what figures, why these backgrounds?

- - Everyone discusses how they felt last week en what icons they need to express this in the body map? How have these feelings to do with the illness and the treatment? (expert by experience in the lead)
  - Everyone goes into the effect of the treatment on their bodies. What icons are used and what is the explanation for these? Where did they put them? How do they deal with treatment burden and can any tips be exchanged? (expert by experience in the lead)

*Wrapping up (10 min)*

- We have made a start. Thank you for contributing. Next week we continue on the same devices. If any questions arise please call us.
- Evaluation of the session.

**Program of the second workshop (1,5 hours)**

*Welcome and recap (10 min)*

- How are you and how was everyones week? Ask consent again for recording.
- Recap last week (10 min), how do you look back at last week?
- Welcoming any new participants.
- Announcing the next four steps that address all kinds of activities and the interference of the treatment. As well as future hopes and dreams. Next to this we will discuss positive effects of the treatment and the people around you.
- Again the breaks are discussed

*Restart body mapping (60 min)*

- Everyone answers the whappbot question which activity they find attractive and what treatment does to this activity. They explain why they choose a certain picture, the meaning to them, where they put it and how they deal with this.
- Everyone answers what they would like to do, but not possible because of the treatment. They explain why they choose a certain picture, the meaning to them, where they put it and how they deal with this (school, jobs, free time, emotions).
- The whappbot question of positive effects of the treatment and discusses this in the group.
- The whappbot question of people in their social network. Which icons did they use, what do they mean, where do they put them on the body map? What can people do to support them?

*Closing (10 min)*

Evaluation

**Program of the third workshop (1,5 hours)**

*Welcome and recap (10 min)*

- How is everyone? How was your week?
- Asking consent for recording
- Recap of last week’s meeting
- Again today four steps of the body map and everyone’s resulting body map
- Breaks are announced.

*Continuation body mapping (45 min)*

- What is it that everyone wants to change in their treatment that would fit treatment better into everyone’s lives. Which icons were chosen, where are they positioned and why?
- Whappbot question what do you do in dealing with treatment? Do they have tips for others? How do they visualize this in the body map?
- Whappbot question what does not always succeed in treatment? How do they visualize this and what does it mean?
- What is it that they want to discuss with care professionals during consultations? How did the body map help to be aware of this issue and raise the question?

*Closing*

- What did you think of the tool as we created it now (15 min)
- Evaluation
- Asking consent for sharing the resulting body maps
- Telephone and mail addresses if questions arise of any kind
- Thanking and reimbursements gift voucher
