# Supplementary material for: Patient and public involvement in the development of the digital tool MyBoT to support communication between young people with a chronic condition and care providers
Source: Health Expect. 2024 Mar 5;27(2):e14003. doi: 10.1111/hex.14003 (PMC10915502; doi:10.1111/hex.14003)
Supplement: Supplementary file 3 — Supporting information. [file HEX-27-e14003-s003.docx]

**Appendix C. Translation of Figure 3**


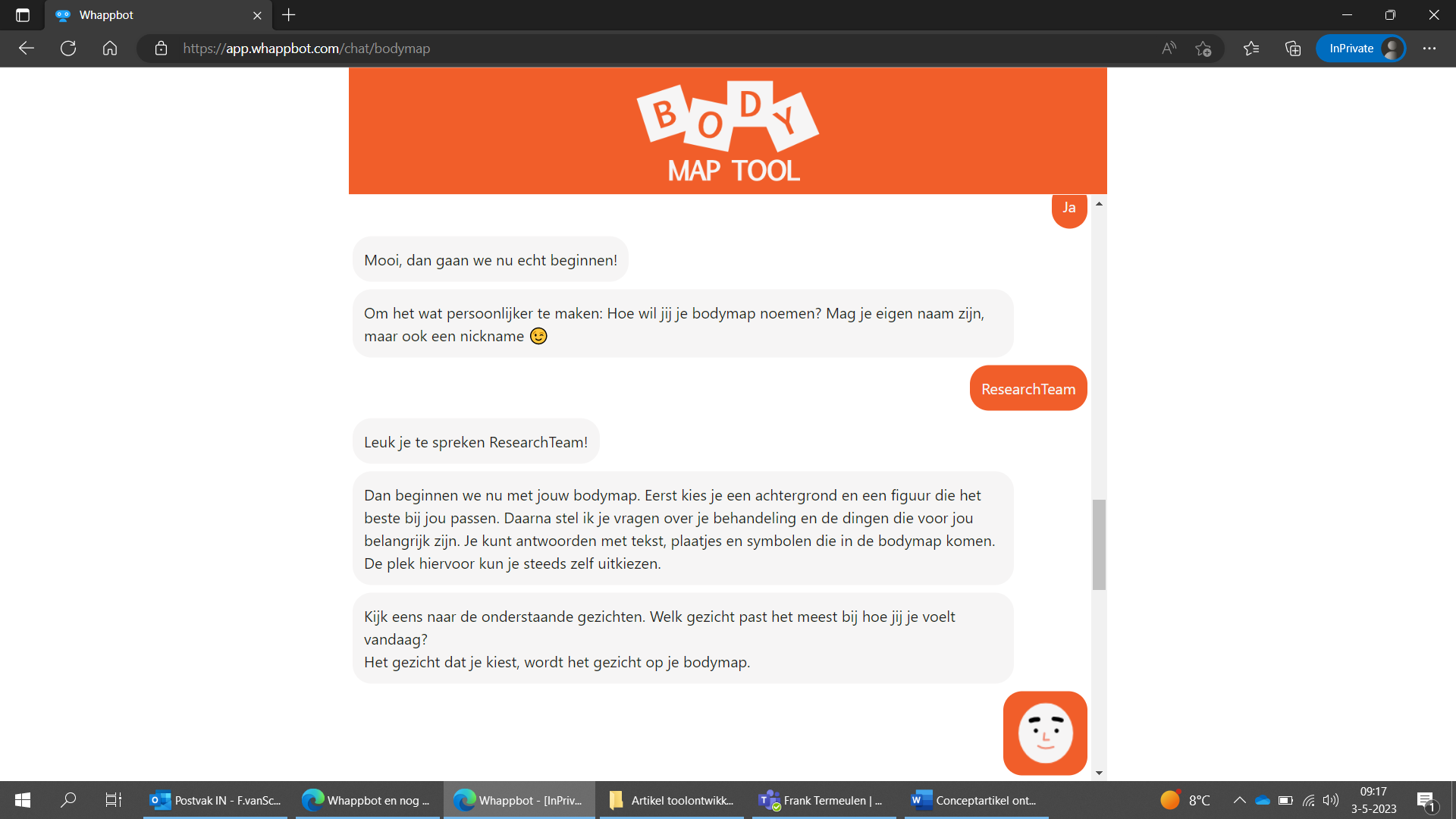


*CHATBOT: Good, let’s start!*

*CHATBOT: To make it a bit more personal: How would you name your body map? Please use a nickname ;)
TOOL USER: ResearchTeam
CHATBOT: Nice to talk to you, ResearchTeam!*

*CHATBOT: Let’s start with your body map. First, you will pick a background and body that suit you best. Then, I will ask questions about your treatment and the things that matter to you. You can answer in text, pictures and symbols that will appear on your body map. You can choose where you wish to put it on your body map.*

*CHATBOT: Take a look at the faces below. Which face best describes how you feel today? The case you choose, will become the face of your body map.
TOOL USER clicks on the happy face.*
